# Perceptions of contraception services among recipients of a combination HIV-prevention interventions for adolescent girls and young women in South Africa: a qualitative study

**DOI:** 10.1186/s12978-020-00970-3

**Published:** 2020-08-14

**Authors:** Kim Jonas, Zoe Duby, Kealeboga Maruping, Janan Dietrich, Nevilene Slingers, Jane Harries, Caroline Kuo, Catherine Mathews

**Affiliations:** 1 Health Systems Research Unit, South African Medical Research Council, Cape Town, South Africa; 2 Adolescent Health Research Unit, Division of Child & Adolescent Psychiatry, University of Cape Town, Cape Town, South Africa; 3 Division of Social and Behavioural Sciences in the School of Public Health and Family Medicine, University of Cape Town, Cape Town, South Africa; 4 Perinatal HIV Research Unit, University of the Witwatersrand, Johannesburg, South Africa; 5 Office of AIDS and TB, South African Medical Research Council, Cape Town, South Africa; 6 Women’s Health Research Unit, School of Public Health and Family Medicine, University of Cape Town, Cape Town, South Africa; 7 Department of Behavioral and Social Sciences, Brown University School of Public Health, Providence, RI USA

**Keywords:** Contraceptives, Contraception services, Access, Contraceptive use, Sexual and reproductive health, Adolescent girls, Young women

## Abstract

**Background:**

Adolescent girls and young women (AGYW) in low- and middle- income countries (LMICs) have high rates of unintended pregnancies and are at higher risk for HIV infection compared to older women of reproductive age. Using a socio-ecological model approach, this research investigated perceptions of contraception services among AGYW who had been recipients of a combination HIV-prevention intervention, to better understand factors affecting their access to and use of contraception services.

**Method:**

Qualitative methods used in this study included focus group discussions (FGDs) and in-depth interviews (IDIs) with 185 AGYW aged 15–24 years living in five of the ten intervention districts. All interviews and FGDs were audio-recorded and data were analyzed thematically using Nvivo 12 software with manual identification of themes and labelling of raw data.

**Results:**

The findings reveal that many AGYW, especially those in the younger age group 15–19 years, experience difficulties in accessing contraception services, mainly at the interpersonal and health service levels. Lack of support for the use of contraceptives from parents/caregivers as well as from sexual partners were key barriers at the interpersonal level; while providers’ negative attitude was the main barrier at the health service level. The majority of school-going AGYW felt that bringing contraception services and other sexual and reproductive health (SRH) services on to the school premises would legitimize their use in the eyes of parents and help to overcome barriers related to parental support and acceptance, as well as overcome some of the health service and structural level barriers. However, views among school-going AGYW about school-based provision of contraception services were mixed, clouded with concerns relating to confidentiality.

**Conclusion:**

Interventions to improve parental/caregiver and sexual partner support for the use of contraception services by AGYW, as well as efforts to expand the provision of contraception services on the school premises are urgently needed. Future interventions should incorporate multi-level approaches to address structural and contextual barriers to access and use of contraception services to gain maximum effect.

## Plain English summary

Adolescents girls and young women (AGYW) in low- and middle- income countries (LMICs) have high rates of unintended pregnancies and are at higher risk for HIV infection compared to older women of reproductive age. To alleviate the rates of unintended pregnancies and reduces the risk for HIV infection among AGYW in South Africa, a combination HIV prevention intervention, funded by the Global Fund was implemented in ten South African districts with a high burden of HIV between 2016 and 2019. The combination HIV prevention intervention aimed to encourage, amongst other things, access to comprehensive HIV, TB and sexual and reproductive health services and commodities such as contraceptives and link AGYW to these services. This study aimed to explore the perspectives of contraception services from AGYW who had been recipients of the combination HIV-prevention intervention. We used an exploratory qualitative research design and applied a socio-ecological model approach to better understand factors affecting their access to and use of contraception services. Data collection method included in-depth interviews (IDIs) and focus group discussions (FGDs) using semi-structured interview guide. AGYW reported to experience difficulties in accessing contraception services. Lack of support for the use of contraceptives from parents/caregivers as well as from sexual partners were key barriers at the interpersonal level; while providers’ negative attitude was the main barrier at the health service level. This study concluded that interventions to improve parental/caregiver and sexual partner support for the use of contraception services by AGYW are urgently needed.

## Background

While the sexual and reproductive healthcare (SRH) needs of adolescent girls and young women (AGYW) have recently gained attention in the global sphere, many of their needs remain unmet [1, 2]. Consequently, approximately 16 million adolescent girls aged 15–19 years give birth each year, contributing to nearly 11% of all births worldwide, and many of these are the result of unintended pregnancies [3, 4]. More than 90% of these births occur in low and middle-income countries (LMICs) [1–4]. In 2013, Sub-Saharan Africa (SSA) was the region with the highest prevalence of teenage pregnancy in the world, accounted for more than 50% of these births [2–5].

Teenage pregnancy is defined as pregnancy occurring among adolescent girls under the age of 20 years, including pregnancy occurring during pre-adolescence, for example a pregnant 12 year old is also referred to as having a teenage pregnancy [3, 4]. Teenage pregnancy is often unintended and unwanted, with undesirable consequences, such as adverse health, educational, social and economic outcomes [3–5]. Despite this, contraceptive uptake among women of reproductive age remains suboptimal. More than 220 million women have an unmet need for contraception in LMICs, with the majority being adolescents in SSA [6]. Furthermore, in SSA, contraceptive use remains low with an estimated unmet need for contraception at 25% among women of reproductive age [5, 6].

In South Africa, about one in five (19%) women of reproductive age (15–49 years) have an unmet need for contraception, with an even higher unmet need for adolescent girls aged 15–19 years at 31% and for AGYW aged 20–24 years at 28% [7]. High unmet need for contraception among AGYW contributes to teenage pregnancy rates which are decreasing at a slower rate in South Africa compared to other developing countries [7, 8]. Although all contraception services are available and offered at no cost from the public health services in South Africa, AGYW are often offered fewer choices for contraceptive methods at public health facilities and given limited explanations of the side effects and mechanism of action, which contributes to the low uptake of contraceptives and subsequently the high unmet need [9–11].

Unintended pregnancy in the South African context is complex with various contributing factors such as poverty, gender inequalities, gender-based violence, inconsistent and incorrect use of contraceptives, poor healthcare provider’s attitudes and behavior, and inadequate SRH information [12–15]. Additionally, young people, especially adolescents in general tend to engage in risky behaviours including sexual activities that eventually put them at risk for unintended pregnancies, amongst other sexually transmitted infections (STI’s), and HIV [16]. Sexual risk-taking behaviours, including early sexual debut, unprotected sex, multiple sex-partners and low and inconsistent contraceptive use are common among young people in South Africa [16–18]. Unintended pregnancy rates among adolescent girls aged 15–19 years have remained unchanged over the past decade and recently are slowly decreasing in the country [7, 19]. This is thought to be attributed to, among other factors, the low contraceptive use to prevent pregnancy, as well as the high unmet need to for contraception among sexually active AGYW [7–11, 19].

To reduce the unmet need for contraceptives and unintended pregnancies among AGYW, improving the availability and accessibility of SRH services for AGYW is necessary. SRH services include, amongst other things, sexual rights and confidential stigma free, unbiased contraception counselling options and services; treatment and prevention of STIs including HIV; and information and counselling services about sexuality. However, the accessibility and use of these services depends on the capacity of the health systems within which these services are delivered. It is critical to ensure that access and use of contraception services for AGYW are aligned with their SRH behaviours, preferences, as well as their reproductive intentions [3–5]. Evidence on what works in reducing teenage pregnancy shows that SRH education, counselling, and provision of contraceptives are effective in increasing adolescent’s knowledge of sexuality and health, contraceptive use, and subsequently decreasing teenage pregnancy [10, 11, 18–21]. Thus, the health system needs to know to the needs of the AGYW in their specific context in order to be responsive, and should have the capacity to deliver the services that meet the SRH needs of AGYW.

**Fig. 1:**
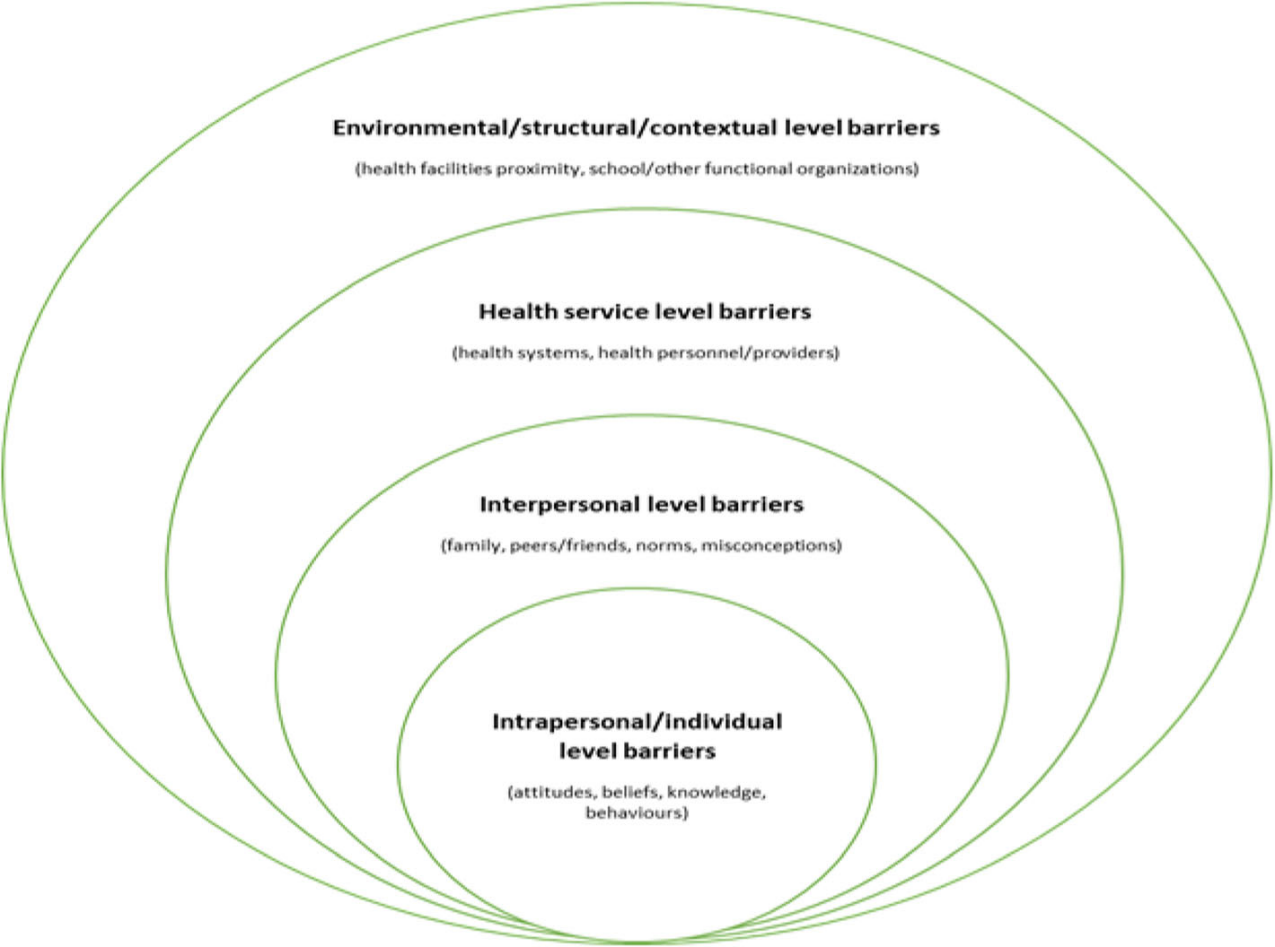
The Social Ecological Model. *Source*: adapted from the Centers for Disease Control and Prevention (CDC), The Social Ecological Model: A Framework for Prevention. https://www.cdc.gov/violenceprevention/publichealthissue/social-ecologicalmodel.html. (Retrieved 23 December 2019)

Contraceptives in South Africa are primarily delivered within public health facilities, with minimal service outreach programmes to communities and schools despite the new Integrated School Health Program’s (ISHP) intention [22], and the health system’s known shortcomings. Additionally, barriers to access and use of contraception services have been identified, including risk perception, poor knowledge of contraceptive methods, lack of support from male partners, as well as other health system’s related challenges. To alleviate these barriers and improve access to SRH services by young people, a combination HIV prevention intervention was implemented between 2016 and 2019. Reducing unintended and unwanted pregnancies among AGYW was one of the five key goals of the combination HIV prevention intervention. The aim of this study therefore, was to explore the perceptions and views of contraception services among AGYW who were recipients of a combination HIV prevention intervention, using a socio-ecological model approach (Fig. 1.).

This model has been used to better understand the health behaviours of individuals as it considers the dynamic interrelationship between an individual and their surroundings is widely accepted [23]. It acknowledges that multilevel factors that include the intrapersonal, interpersonal, community, and societal levels shape an individual’s behavior [23]. For example, AGYW’s intrapersonal (individual) factors such as knowledge of contraceptives can influence her behaviour to access, or not access contraception services. The model has been applied in similar studies, such as studies investigating perceptions of healthcare providers providing SRH services to refugee and migrant women [24, 25]. Therefore, we used this model to gain a holistic view of the barriers and facilitators for AGYW in accessing contraception services and adapted the model to demonstrate health services and environmental (contextual) related level barriers, as specific to this study. This holistic understanding of the factors affecting AGYW’s access and use of contraception services could be useful in improving future interventions that aim to prevent pregnancy among this sub-population and help improve uptake on contraceptives.

## Methods

This study is part of a larger study, an evaluation of the combination HIV prevention intervention, which includes a qualitative component to explore the perceived impact of the intervention on the cognitions, behaviour, and social environments of AGYW who participated in the intervention. The combination HIV prevention intervention was implemented in ten South African districts with a high burden of HIV between 2016 and 2019. The ten districts were: Bojanala, (North West Province), City of Cape Town (Western Cape Province), Ehlanzeni (Mpumalanga), Gert Sibande (Mpumalanga Province), Greater Sekhukhune (Limpopo Province), OR Tambo (Eastern Cape), Nelson Mandela Bay (Eastern Cape), Tshwane (Gauteng Province), King Cetshwayo and Zululand (KwaZulu-Natal Province). The combination HIV prevention intervention aimed to encourage, amongst other things, access to comprehensive HIV, Tuberculosis (TB) and SRH services and commodities and rights-based SRH education and link AGYW to these services. This study focuses on the linkage and access to contraception services, which are provided in the public health facilities.

### Study design and setting

This qualitative study was conducted among AGYW who were recipients of the combination HIV prevention intervention, living in five of the 10 intervention districts in South Africa. The five districts selected for this study included: Bojanala, City of Cape Town, Gert Sibande, Nelson Mandela Bay, and King Cetshwayo. The five districts were purposively selected, as well as two schools from a list of schools where the intervention was being implemented. For out-of-school intervention recipients, one Rise Club was purposively selected from the five intervention districts. Rise Clubs were constituted by 15–20 young women who met regularly or at least once a month to discuss issues that affected them, following a curriculum contained in Rise Magazines. They were led by a trained facilitator who provided SRH education, and linked the young women to health services, including HIV testing and services, educational, and to economic opportunities and local microenterprise development organisations. The clubs also linked young women to career guidance through career jamborees and homework support. The Clubs were implemented for in-school adolescent girls aged 15–19 years, and for out-of-school young women aged 20–24-years. A detailed description of the combination HIV prevention intervention is included in Box 1. Participants were recruited and invited to participate in the study by a team of researchers with the assistance of the intervention implementers and school liaison teachers.

A qualitative descriptive study design was employed in this study. This design is applied when a study seeks to discover and describe a phenomenon, a process, or perspectives and views of the people involved and offer comprehensive information of an event [26–28]. The design also provide means to voice the views and opinions of the participants and is useful in studies that aim to improve health care service design and delivery such as the contraception services, and can influence health service provision through recommendations and policy changes [29, 30].

### Sampling

The sampling framework consisted of districts, schools, and Rise Clubs out-of-school within the districts where the combination prevention intervention was implemented in five provinces of South Africa. In South Africa there are nine provinces containing 52 districts; the intervention was implemented in 10 high-burdened districts in terms of the HIV epidemic, within seven provinces of the country. We selected five of the 10 districts within five provinces; the selection was based on the convenience and feasibility to conduct the study (e.g., in terms of logistics). The districts represented semi-urban, rural and urban settings.

Within each school, we conducted up to three focus group discussions (FGDs) with intervention recipients, including 6–10 AGYW per group, two sets of serial in-depth interviews (SIDIs), or one-on-one in-depth interviews (maximum of three) per district. The interviews and FGDs were conducted by an experienced FGD facilitator, with assistance from a note-taker. FGD groups were stratified into different age groups 15–18 years and 19–24 years.

### Participants

AGYW between the ages of 15–24 years, who took part in the combination intervention components were recruited from the schools and the out-of-school Rise clubs within the districts. Participants for the SIDIs were identified from the FGDs as participants that could share more opinions and views outside a bigger group on specific issues surrounding contraception services. One-time one-on-one in-depth interviews were used when it was not possible to form a group of 6–10 AGYW. Thus, a total of 19 FGDs, 53 SIDIs and 4 IDIs comprising of 185 AGYW who were intervention recipients were included in this study.

### Data collection tools

An open-ended semi-structured interview guide with probes was used to explore AGYW’s perceptions of contraception services as means to gain an understanding of their perceived barriers and enablers to accessing the services. We asked AGYW questions related to health services access and use, an example: *We are interested in hearing from you about the health services you and your friends receive at school and in the community. We are especially interested in health services that focus on HIV, TB, and other STIs, teen pregnancy, could you please tell us what you think of these services? Probe questions: To what extent do these services meet you and your friends’ sexual and reproductive health needs, for example contraceptive needs? Please elaborate. What makes it easy or difficult for young women and girls like you to access contraceptives? Please elaborate*.

All interviews and discussions were conducted between August 2018 and March 2019, in English, or isiXhosa, or isiZulu, or Setswana, audio-recorded, and hand-written notes were taken during the discussions, which lasted about 30 min to an hour. A brief questionnaire to document AGYW’s socio-demographic characteristics was also administered.

### Procedure

Two researchers, (KJ and KM) together with research assistants, facilitated the group discussions and conducted the SIDIs and one-time one-on-one individual in-depth interviews with AGYW. Both researchers are experienced Black African female researchers with one having obtained a PhD (KJ) and the other one an Honours degree (KM) at the time of data collection. The data collection team spoke one or two of the native languages spoken in the intervention districts other than English. The race and languages spoken by the data collection team and that of the AGYW was matched purposely to ensure the team was accepted, trusted, understood and in turn could understand the nuances of what was said during the interviews. There were no existing relationships with participants prior to data collection for this study and therefore no conflict of interest between the participants and the researchers. All interviews and FGDs were conducted in the schools or in a pre-arranged venue for the out-of-school AGYW.

Data saturation, where no new topics or issues came up during data collection, was discussed after the 15th FGD. It became clear that there were no new views transpiring from the discussions by the 19th FGD and data was deemed saturated at this point with regards to the FGDs. With regards to SIDIs, three interviews were conducted with each selected AGYW with a focus on a specific issue such as views on contraceptive use by AGYW which was discussed extensively and often repeated in all three interviews until there was no new information transpiring. At this point data were deemed saturated and interviews would end. Data triangulation was also conducted utilizing written notes and observations during data collection.

### Data analysis

Data were analysed using thematic analysis. The data analysis followed the Tesch’s eight steps for coding and analysing qualitative data [31]. Data were transcribed verbatim from the audiotape recordings, reviewed by the interviewer for accuracy, then translated to English if they were conducted in another language, and reviewed again to ensure content was not lost in translation, and then finalized for analysis. Typed transcripts were read, and codes were developed and defined based on the objectives of the study, thus themes were derived from the data (example of the coding process and data synthesis is shown in Table 1.).

**Table 1:** Example of coding process and data synthesis of the perceptions of contraception services among AGYW who were recipients of a combination HIV prevention intervention in five districts of South Africa

Quote (meaning unit)	Theme	Sub-theme	^**a**^SEM level
Contraceptives are very important you see…Because if you don’t use contraceptives you may fall pregnant	Importance of using contraceptives	Perceived benefits of contraceptive use	Intrapersonal
It is important because what if you get raped while walking, get kidnapped and raped, there is a difference in what happens because you will be safe from being pregnant even though you can have STI’s and HIV			Intrapersonal Interpersonal Contextual
I have some information. I also get from LO [Life Orientation] however it is not enough I still need some more information, for example, I don’t know the different types of contraceptives and how they work on your body	Barriers to contraceptive access and use	Lack of information	intrapersonal
To be honest, we are not sure but we know that the injection can prevent you from being pregnant, we don’t have much knowledge about the other issues like the fact that it makes your body to hang, the gaining of weight, and the issue of being wet			Intrapersonal Interpersonal
The clinic is far…sometimes you don’t get assisted if you go after school, and then you have to go the next day and be absent at school		Facility distance	Health services Structural/ environmental
The needle makes other people gain weight, it makes others lose weight. Out there people will start to gossip about you and say you have AIDS, you have TB you are sick and stuff		Myths and Misconception	Interpersonal Contextual
Some clinics can be frustrating like you would go to a clinic for contraception, the nurses will start asking all sorts of question; why are you here? Young as you are! Do you have a boyfriend? And because of these questions and that you feel embarrassed you end up leaving without accessing the services		Provider attitude	Health services
My boyfriend doesn’t know that I am on contraceptives I hide it from him, because if he found out, I don’t know what would happen because he doesn’t approve of me taking the injection		Lack of male partner support	Interpersonal
I don’t think my mother would understand if I talk about going to prevent (family planning). She would think that I’m planning to have sex or that I have slept with a boy that’s why I want to go prevent or if I want to check for HIV she’d think that I’ve had sex with a boy		Lack of parental support	Interpersonal Contextual
For most of us here we live nearby so the day hospital is a walking distance so its easy for us to get condoms and family planning and its free also	Factors enabling access	Clinic/ service provider proximity	Health services
I talk to my mom, she even reminds me of the next visit date for family planning, and she tells me that just because I have my first child		Parental/caregiver support	Interpersonal
He also tells me that I have to prevent because I am in school. I cannot afford another child. My future will get ruined if I am busy having children. I have to prevent.		Male partner support	Interpersonal
But as for me, there was a time when I was using tablets… Then 1 day when I went to the clinic to fetch them, the nurse told me that an injection is much better than tablets because with tablets, you are expected to drink them everyday… But, with injection, when you use it, you are again expected to come after 3 months for a repeat… So, she discussed it with me and made me to understand the difference between the injection and the tablet, unlike other nurses who do not have time to explain, they just give you what you said you wanted.		Provider positive attitude	Health services
Like contraceptive, I did not know the different types but now I have a little bit of knowledge about them	Influence of the intervention	Gained information	Intrapersonal
At first, we were using only condom, but after we joined Rise [Rise Club] they said things like preventing, which is good... for me not to be pregnant. So now I know when I have to have sex, I must use a condom so to protect myself and prevent [using contraceptives]			Intrapersonal Interpersonal
I think it has changed my life because my mom did not want me to prevent, so I joined Rise [Rise Club] and explained to her what they told us there. So she let’s me prevent now		Parental support	Interpersonal

^a^*SEM* Socio-ecological model

A team of researchers, independent to the interviews, developed and checked the coding until they reached consensus. Then, codes were grouped into sub-themes and then into themes. Data were coded and analysed using Nvivo 12 qualitative data analysis software. The coded transcripts were analysed by running query reports and primary document tables of codes by theme, to explore the issues from the various discussions. The study team met regularly to compare and discuss findings until consensus was reached.

### Trustworthiness

Triangulation of findings from different data sources (AGYW 15–18 years old group, AGYW 19–24 years old group, research assistants’ notes, interviewer observations), and data methods (FGDs, SIDIs, IDIs, feedback workshops) were used to increase trustworthiness of our findings. Field notes, debrief sessions, and data collection team reflections between the field team and the larger study team were conducted regularly to discuss themes coded from the data. The transcription-translation process of raw data was performed by the research assistants, reviewed and quality checked by the two independent researchers, and were ultimately reviewed by the authors, keeping an audit trail to help validate the coding process and data analysis.

## Findings

The findings of this study are presented below starting with the brief descriptive characteristics of the participants. Thereafter, themes are presented using the socio-ecological levels model encompassing individual or intrapersonal, interpersonal, health service levels, and environmental or contextual level barriers. The quotes are translations of raw data with descriptive details of the participant citing the quotes in bold. It is worth noting that some of the factors could be observed in more than one level as the interrelationships are complex.

### Participants

Among the 185 AGYW participants, 154 (83.2%) were enrolled in secondary schools, between Grades 8–11. Among those not enrolled in school, 22 (71.0%) had completed secondary school. The majority of participants self-identified as Black Africans with only 8.6% self-identified as Coloured. Most participants were between the ages of 15–19 years (147, 79.5%) with 165 (89.2%) currently living with their parents (mother/father). No one reported living alone. Almost all AGYW were receiving and or living in the household where the social grant (approximately between 29 USD for the child support grant and 111 USD for the old age grant) was the main source of financial support. Among the AGYW, 49 (26.5%) were taking care of a child or other family members in the household. The majority of the AGYW reported to have never been pregnant before, while 38 (20.5%) reported to have been pregnant before.

### Intrapersonal / individual level barriers and facilitators

#### Barriers to access and use of contraception services

Lack of knowledge about contraception services was perceived to be one of the barriers preventing AGYW from accessing the services. The majority of AGYW reported that they needed more information about contraceptives because sometimes at the clinic they do not explain to them how the injection works and what side effects to expects, as well as advantages and disadvantages of using the injection. Of particular interest was the small number of methods of contraception mentioned by the AGYW as almost all of them mentioned the injection while a few mentioned the implant, with no other methods mentioned.

I have some information. I also get from LO [Life Orientation]; however, it is not enough I still need some more information, for example, I don’t know the different types of contraceptives and how they work on your body… **AGYW 15-18 years, FGD, KZN**

To be honest, we are not sure but we know that the injection can prevent you from being pregnant, we don’t have much knowledge about the other issues like the fact that it makes your body to hang, the gaining of weight, and the issue of being wet. **AGYW 15-18 years, FGD, WC**

We don’t have enough information, we have little information… **AGYW 15–18 years, FGD, WC**

#### Facilitators/enablers to access and use of contraception services

The majority of the AGYW perceived the use of contraception services as important, relating sentiments from “it is important to use contraceptives” to “it’s the right thing to do”. AGYW also added that using contraceptives is important to prevent young girls from falling pregnant while they are still in school.

I also support it because some of the girls are sexually active you see, so they become scared of speaking to their mothers in terms of what they should do, what must I do, should I use a condom or not. So, using contraceptives assist them with preventing teenage pregnancy. **AGYW 15-18 years, FGD, WC**

Contraceptives are very important you see…Because if you don’t use contraceptives you may fall pregnant… **AGYW 15-18 years, SIDI, WC**

### Interpersonal level barriers and facilitators

#### Interpersonal barriers to access and use of contraception services

Myths and misconceptions were often cited by AGYW as one of the barriers preventing them from accessing contraception services. AGYW perceived the injectable contraceptive to be not good for them as it affects their body weight and shape and that people will make other conclusions about the change in body weight. The perceived side effects or body changes due to the use of the contraceptive injection are also informed by the contextual factors such as social norms around body weight. The perception that contraceptive use affects fertility was also reported by AGYW stating that they fear not being able to have children in the future due to the use of contraceptives.

The needle makes other people gain weight, it makes others lose weight. Out there people will start to gossip about you and say you have AIDS, you have TB you are sick and stuff. **AGYW 15-18 years, FGD, MP**

Some say contraceptives are going to ruin their wombs and, in the future, they will not be able to have kids, so they decide not to use contraceptives because they would like to have kids in the future. **AGYW 15-18 years, FGD, EC**

Lack of support for the use of contraceptives from parents was commonly reported by AGYW, who felt that their parents did not understand why young girls should use contraception unless they are planning to have sex. With regards to support from male partners for the use of contraceptive services, some AGYW stated that they hide their use of contraceptives from their boyfriends as they do not support it. AGYW also reported that their boyfriends stated clearly that they do not want them to use contraceptives because of the myths and misconceptions around contraceptive use, such as becoming infertile in the future and “decreased libido” and therefore they do not discuss anything to do with contraception as partners. General fear of being seen at the clinic for contraception services and other privacy issues were also cited a number of times by AGYW, stating that it is difficult for them and other AGYW to just visit the clinic for these services.

I don’t think my mother would understand if I talk about going to prevent (family planning). She would think that I’m planning to have sex or that I have slept with a boy that’s why I want to go prevent or if I want to check for HIV she’d think that I’ve had sex with a boy… **AGYW 15-18 years, FGD, EC**

No. Grandmother was totally against it... She says that these things may have negative impact where a person can’t conceive in the near future or whatever, so that’s why I can’t use them. **AGYW 15-18 years, SIDI, EC**

My boyfriend doesn’t know that I am on contraceptives I hide it from him, because if he found out, I don’t know what would happen because he doesn’t approve of me taking the injection… **AGYW 15-18 years, FGD, MP**

#### Interpersonal facilitators/enablers to access and use of contraception services

Parental and partner support were perceived as factors enabling access to contraception services by AGYW. They reported when their parents and partners know about them using contraceptives, and support their use, it becomes easier to use and access them correctly and consistently.

I talk to my mom, she even reminds me of the next visit date for family planning, and she tells me that just because I have my first child… **AGYW 15-18 years, FGD, MP**

He also tells me that I have to prevent because I am in school. I cannot afford another child. My future will get ruined if I am busy having children. I have to prevent. **AGYW 15-18 years, SIDI, MP**

### Health service level barriers and facilitators

#### Health services’ barriers to access and use of contraception services

Health service provider’s negative attitudes were cited as the main perceived barrier to accessing the services. AGYW stated that nurses shout at them saying they are too young to be using contraceptives and humiliate them by asking questions in front of everyone at the clinic. They perceived the negative attitude of providers as the main reason many of them do not use contraceptives or are afraid to access the services.

Some clinics can be frustrating like you would go to a clinic for contraception, the nurses will start asking all sorts of question; why are you here? Young as you are! Do you have a boyfriend? And because of these questions and that you feel embarrassed you end up leaving without accessing the services. **AGYW 15-18 years, FGD, MP**

I agree with what she is saying regarding nurses not being able to talk to people nicely, they can’t. A nurse will utter something that will be so hurtful, when you speak and tell them you are here to get contraceptives, they won’t speak to you privately in a room instead they will loudly say why are you here for contraceptives in front of people and you can imagine how many people are at the clinic. They will make noise and say why are you here for contraceptives when you are so young, why? You like boys. **AGYW 15-18 years, FGD, WC**

#### Health services’ facilitators/enablers to access and use of contraception services

A provider’s positive attitude was also perceived as an enabling factor to accessing contraception services. AGYW stated that the positive and welcoming attitude of some nurses when they visit the clinic encourages them to continue coming back for contraception services. AGYW described the pleasure they feel when nurses are happy to see them coming for contraceptives and that further motivates them to come for the services. AGYW also reported being appreciative when the nurses discuss the contraceptive methods with them and make them feel comfortable with using the prescribed method.

But as for me, there was a time when I was using tablets… Then one day when I went to the clinic to fetch them, the nurse told me that an injection is much better than tablets because with tablets, you are expected to drink them everyday… But, with injection, when you use it, you are again expected to come after three months for a repeat… So, she discussed it with me and made me to understand the difference between the injection and the tablet, unlike other nurses who do not have time to explain, they just give you what you said you wanted.” **AGYW 19-24 years, SIDI, KZN**

Furthermore, AGYW stated that receiving quick service is something they appreciate and encourages them to come back next time as they know that they will not spend hours standing in the queue.

A quick service because sometimes you are just there for prevention…. **AGYW 15-18 years, FGD, MP**

### Environmental/structural/contextual level barriers

#### Environmental/contextual barriers to access and use of contraception services

With regards to the environmental or structural barriers, the societal challenges such as violence and rape were common for AGYW. As such, almost all AGYW supported the use of contraceptives to prevent pregnancy in case of unforeseen circumstances, making reference to “in case someone gets raped”.

It is important because what if you get raped while walking, get kidnapped and raped, there is a difference in what happens because you will be safe from being pregnant even though you can have STIs and HIV. **AGYW 15-18 years, FGD, MP**

Structural factors such as clinic or facility distance were also discussed by AGYW, particularly by the AGYW residing in rural settings stating that some clinics are far, and you need the whole day to get services at the facilities. AGYW in the rural settings added that they need to be absent from school when they need to access contraception services.

Some clinics are far, you need to use transport to get to them and parents complain about not having money to give them so that they can go to the clinic… **AGYW, 15-19 years, FGD, KZN**

The clinic is far…sometimes you don’t get assisted if you go after school, and then you have to go the next day and be absent at school. **AGYW, 20-24 years, FGD, MP**

Additionally, views on the school’s environment as the place to provide contraceptive services varied among AGYW. Some AGYW thought that teachers at school will humiliate them in front of other learners disclosing their use of contraceptives if they see them going to the room where the services are being provided in the school. Others expressed concerns around confidentiality in the school as many will see them and their parents will find out. Concerns about the school nurse or counsellor being affiliated with the school were also expressed by AGYW and suggested that someone unknown in the school and in the community the school is based in may be a better option.

Aah, I don’t think it will be easy, because they are afraid that people will talk and when they talk, the talk might end up reaching their homes… Because not everyone can be trusted, especially here at school… I don’t see them getting contraceptives here at school where everyone is looking. **AGYW 15-18 years, FGD, MP**

#### Environmental/ contextual facilitators/enablers to access and use of contraception services

The clinic’s proximity was reported as one of the factors enabling access to contraception services by some AGYW. They stated that it is easy for them to go to the clinic for contraceptives as the clinic is nearby their school.

For most of us here we live nearby so the day hospital is a walking distance so it’s easy for us to get condoms and family planning and its free also. **AGYW 15-18 years, FGD, WC**

Most AGYW thought that it would be useful if contraception services could be provided within school premises, while others thought it would not work as they thought that teachers will know about it and gossip about them using contraceptives.

I also think that would be a great vision because other children are scared to talk to their parents, mom at school they are discussing contraceptives… maybe if the nurses are available, they will get contraceptives. And then when they get home, they can tell their mother that at school we were given contraceptives because of this and that… **AGYW 15-18 years, FGD, WC**

That would be a good idea [to bring family planning into schools], many of them would do it because maybe some of them are afraid to go to the clinic because they get scolded… **AGYW 15-18 years, SIDI, EC**

It’s not easy for some children to leave their home and go to the clinic because they can’t ask their parents to go to the clinic but here at school it would be easier for them to get help. **AGYW 15-19 years, FGD, KZN**

#### Influence of the combination intervention on AGYW access and use of contraceptives

When the AGYW were asked about how they perceived the intervention to have impacted on their lives, most of them stated that the intervention helped them gain better knowledge of contraceptives and the importance of using them. Some AGYW stated that being part of the intervention enabled them to better communicate SRH information to their parents and or caregivers, making them understand why it would be important for them to use contraceptives, citing reasons provided to them during the intervention sessions.

Like contraceptive, I did not know the different types but now I have a little bit of knowledge about them **AGYW 15-18 years, FGD, EC**

We have learnt like, when like you using contraceptives it helps you like not to fall pregnant **AGYW 15-18 years, FGD, WC**

I think it has changed my life because my mom did not want me to prevent, so I joined Rise [Rise Clubs] and explained to her what they told us there. So she let's me prevent now… **AGYW 15-18 years, FGD, WC**

The AGYW also stated that being part of the intervention made it easier for them to access contraception services as they gained confidence and understanding of the services during the intervention sessions. Some reported to find it even more helpful in helping them understanding why it is important to use contraceptives over and above using a condom when having sex.

At first, we were using only condom, but after we joined Rise [Rise Clubs] they said things like preventing, which is good… for me not to be pregnant. So now I know when I have to have sex, I must use a condom so to protect myself and prevent [using contraceptives]… **AGYW 19-24 years, FGD, KZN**

Rise [Rise Clubs] has changed me because I wasn't preventing [not using contraceptives], and then after we were told that you can be pregnant while using a condom because it can burst. Then I went to prevent [use contraceptives]. **AGYW 15-18 years, FGD, WC**

## Discussion

This study sought to explore perceptions of contraception services among AGYW who were exposed to the combination HIV prevention intervention in order to identify barriers and enablers for contraceptive access and use as the intervention aimed to encourage access and linkage to these services. At the intrapersonal or individual level, lack of knowledge about the different types of contraceptives and how they work was one of the main barriers to access and use of contraceptives according to the AGYW in this study. Limited knowledge of SRH, including contraception services among AGYW is not uncommon as it has been previously reported to contribute to poor access and use of the services [8, 32–34]. Furthermore, this finding complements previous research among health workers providing contraception services to AGYW where health providers reported that AGYW need more information on SRH and counselling around family planning options including condom use [35].

Thus, efforts to improve knowledge among AGYW pertaining to contraception services requires urgent attention to improve access and use of the services, and ultimately alleviate unintended and unwanted pregnancies among this subpopulation. It is noteworthy that a substantial proportion of AGYW in this study viewed the use of contraceptives positively and important to use; however, limited knowledge hindered their potential to fully access and use the services. Therefore, it is crucial to leverage this window of opportunity by escalating measures to increase SRH knowledge and information services for AGYW and promote the use of contraceptives. Additionally, this will help dismantle the myths and misconceptions around contraceptives which also came up as a serious barrier, and thereby improve the access and use of the services.

Partner support for the use of modern contraceptives has been a long-standing challenge in improving contraceptive uptake among women of reproductive age, particularly in the developing countries and is a cause for concern [8, 11, 36–39]. Engaging partners, particularly male partners in SRH services has been shown to improve men’s knowledge and attitudes towards the use of contraceptives by their female partners [38–44]. Therefore, efforts to involve male partners in SRH interventions need to be accelerated to improve access and use of contraceptives by AGYW.

According to the AGYW, being part of the intervention helped improve their knowledge about contraceptives and improved their self-esteem, which enabled them to access contraception services. At the interpersonal level, lack of support from parents and partners was a common theme in addition to the myths and misconceptions. It is unclear if parents do not support the use of contraceptives among AGYW in this study because of the myths and misconception and community norms around contraceptive use, or because of the thought that if their children are using contraceptives it will mean that they are sexually active and the do not approve of that. Although literature around parent-child interactions relating to SRH is inconclusive in some instances [43], there is increasing evidence demonstrating the influence of parental support on positive SRH behaviours. Research conducted in high income countries found that limited parental monitoring and support were associated with sexual risk behaviour such as condomless sex, while parental support and knowledge of adolescent’s sexual activity were positively associated with contraceptive use, and delay of sexual debut, among other things [45, 46].

Furthermore, lack of information and parental support among adolescent mothers were amongst the barriers to postpartum contraceptive use [46]. Those AGYW whose parents and/or partners were aware of their contraceptive use in this study provided support and motivation, including reminders to attend follow-up appointments. Moreover, being part of the intervention enabled the AGYW to communicate with their parents about SRH issues, including contraceptives and made it easy for them to explain to their parents why it is important for them to use contraceptives. This communication was reported to result in AGYW being permitted and given support by the parent/s to use contraceptives. Evidently, to improve contraceptive access and use among AGYW requires the involvement of parents and partners, including all supportive structures of AGYW as recommended by Harper et al., (2004) [47]. Therefore, integrating SRH programs into AGYW’s supportive networks covering the broad socio-ecological levels within which they live in may be a step toward improving contraceptive coverage and effective use, and thus reducing the unmet need for contraception among this subpopulation.

Providers’ negative attitudes in the public health facilities were commonly cited for the health services level barriers. This finding is not surprising as health providers’ negative attitudes have been documented to affect access and use of contraceptives among AGYW. Negative attitudes of health providers have been extensively documented and contribute to poor access and use of SRH services at large, and by adolescent girls in particular [48–56]. Jonas et al., (2019) also reported on the health service level of barriers to access and use of contraceptives as narrated by nurses providing family planning services to adolescent girls in South Africa whereby nurses reported to assume that if an adolescent girl is coming for contraceptives it means she has a boyfriend and thus warranting negative behaviour towards the girl as she is “still too young” to be having boyfriends [34]. Amongst the facilitators for access and use of contraceptives by AGYW, health providers’ positive attitude was frequently mentioned. Also, the relationship between intervention facilitators and clinics’ staff appeared to facilitate access and use of contraception services according to the AGYW, as intervention facilitators engaged with nurses about the intervention and its objectives, to enhance linkages to care. It is crucial therefore, to escalate efforts aimed at improving health providers’ attitudes towards AGYW accessing contraception services and engage them in interventions for AGYW’s SRH. Interventions, such as sensitisation and value clarification trainings to help health providers manage their personal values and morals regarding AGYW’s sexuality and contraceptives, and aligning these with reality, maybe of great help in averting negative attitudes of health providers in contraception services. Application of such interventions have yielded positive outcomes where negative attitudes of health providers towards abortion services and providing contraception services to adolescent girls were averted in South African context and other similar context [57, 58].

There are some important limitations in interpreting the findings of this study. One such limitation is the purposive sampling approach, both for the districts and AGYW included in this study which may potentially bring bias to our findings and limits generalizability. We also did not ask about religious or cultural affiliations from the AGYW, future studies may need to explore this aspect to get more insights of the influence of religion and culture on AGYW perceptions of contraceptives. However, despite these limitations, our findings emphasize the seriousness of the need to address the barriers to access and use of contraception services and offer alternative approaches to realizing contraception coverage among AGYW in South Africa.

## Conclusion

AGYW in South Africa experience difficulties in accessing contraception services mainly at the interpersonal and health service levels. Lack of support for the use of contraceptives from parents/caregivers as well as from sexual partners were key barriers at the interpersonal level; while a provider’s negative attitude was the main barrier at the health service level. Views among school-going AGYW regarding the provision of contraception services on the school premises were positive but concerns around privacy and trust issues that AGYW have about teachers and other learners weakened the feelings. The combination HIV prevention intervention appeared to have helped AGYW as they reported to have gained knowledge about contraceptives, improved the ability to communicate with parents/caregivers about SRH related topics and increased confidence in accessing contraception services. Interventions to improve parental/caregiver and partner support for the use of contraception services by AGYW, as well as efforts to expand the provision of contraception services on the school premises are urgently needed. Improved access to and use of contraception services will enable AGYW to control their fertility, maximize educational and economic opportunities, and enhance their SRH and wellbeing. This in turn, will reduce the unmet need for contraception and decrease the unintended and unwanted pregnancies among AGYW. Future interventions should incorporate multi-level approaches in addressing structural and contextual barriers to access and use of contraception services to gain maximum effect.

### Recommendations

At the individual level, efforts to improve AGYW knowledge and information about contraception services should be strengthened. This can be done through comprehensive sexuality education inside and outside the classroom. Resources containing SRH information, such as myth busters and contraceptive method-specific information pamphlets and posters should be freely available and displayed in major youth-friendly zones, this way AGYW have the information at their disposal and can access it when necessary. More research is needed to uncover the issues around the lack of parental and sexual partner support for the use of contraceptives for the interpersonal level barriers. Community gatherings, church or other religious activities, and sports and recreational facilities and activities should be used to offer educational interventions and promote the use of contraceptives by AGYW as all stakeholders will be found in these settings. The interventions should address community norms and the myths around contraceptive use and the perceived future fertility decrease, as well as highlight the impact of unintended and unwanted pregnancy during adolescence with parents. Addressing the health services level barriers will require an entire health systems transformation where providers’ attitudes are improved, only competent youth-friendly providers are serving AGYW, responsive and time sensitive services are provided to AGYW, and prioritization and effective implementation of SRH policies for the youth are ensured. This requires a strong political will to advocate for and ensure availability of resources, including human resources for health for AGYW to fully access and use contraception services.

## Data Availability

The dataset used for the current study is available from the corresponding author on reasonable request.

## Abbreviations

AGYW: Adolescent Girls and Young Women
EC: Eastern Cape
FGD: Focus Group Discussion
HIV: Human Immunodeficiency Virus
IDI: In-Depth Interview
ISHP: Integrated School Health Program
KZN: KwaZulu Natal
LMIC: Low- and- Middle- Income Country
MP: Mpumalanga
SIDI: Serial In-Depth Interveiw
SRH: Sexual and Reproductive Health
SSA: Sub-Saharan Africa
STI: Sexually Transmitted Infection
TB: Tuberculosis
USD: United States Dollar
WC: Western Cape
